# CONSIDERING AN INTERDISCIPLINARY DEMENTIA INTERVENTION, COMBINING BEHAVIORAL AND OCCUPATIONAL THERAPY

**DOI:** 10.1093/geroni/igz038.506

**Published:** 2019-11-08

**Authors:** Lou Frankenstein

**Affiliations:** Chemnitz University of Technology, Institut for Applied Gerontopsychology and Cognition, Germany, Chemnitz, Germany

## Abstract

Behavioral therapy for people diagnosed with dementia and their informal caregivers can enhance the quality of life and the accomplishment of daily routines. The effectiveness of occupational therapy in dementia has also been proven several times. Still those therapies are often not part of the regular treatment. Through literature analyses and three focus group discussions with experts of both professions four occupational (ESP, WHEDA, Ergodem and HED-I) and two behavioral interventions (CBTAC, cordial-program) were compared to clarify how much and in what ways behavioral and occupational therapy in dementia overlap and differ. The interventions are similar with respect to intervention characteristics: They are non-pharmacological, client-centered, put a major emphasis on involving the caregivers and are similarly structured. Whilst occupational programs focus on practical issues, such as empowerment to perform daily activities or adjustment of the environment, cognitive-behavioral interventions specialize on planning activities, communication and reminiscence. These differences result from the theoretical basis and the primary goals of the professions. Behavioral therapy developed from learning theories and cognitive techniques. In contrast occupational therapy is based on environmental theories and the idea of empowerment. In addition two activating programs were included (CST, MAKS-active) to promote physical activity and social interactions since both positively influence the course of the disease. The focus group participants supported the idea of an interdisciplinary cooperation. The results suggest, that an intervention offered by both professions, ideally in cooperation, encompassing key elements identified in the studied programs would be an advance in dementia therapy.

